# sEMG-Based Natural Control Interface for a Variable Stiffness Transradial Hand Prosthesis

**DOI:** 10.3389/fnbot.2022.789341

**Published:** 2022-03-11

**Authors:** Elif Hocaoglu, Volkan Patoglu

**Affiliations:** ^1^Faculty of Engineering and Natural Sciences, Sabancı University, Istanbul, Turkey; ^2^School of Engineering and Natural Sciences, Istanbul Medipol University, Istanbul, Turkey

**Keywords:** tele-impedance control, sEMG-based control interface, variable stiffness actuation, transradial hand prosthesis, impedance modulation

## Abstract

We propose, implement, and evaluate a natural human-machine control interface for a variable stiffness transradial hand prosthesis that achieves tele-impedance control through surface electromyography (sEMG) signals. This interface, together with variable stiffness actuation (VSA), enables an amputee to modulate the impedance of the prosthetic limb to properly match the requirements of a task while performing activities of daily living (ADL). Both the desired position and stiffness references are estimated through sEMG signals and used to control the VSA hand prosthesis. In particular, regulation of hand impedance is managed through the impedance measurements of the intact upper arm; this control takes place naturally and automatically as the amputee interacts with the environment, while the position of the hand prosthesis is regulated intentionally by the amputee through the estimated position of the shoulder. The proposed approach is advantageous since the impedance regulation takes place naturally without requiring amputees' attention and diminishing their functional capability. Consequently, the proposed interface is easy to use, does not require long training periods or interferes with the control of intact body segments. This control approach is evaluated through human subject experiments conducted over able volunteers where adequate estimation of references and independent control of position and stiffness are demonstrated.

## 1. Introduction

According to the WHO, there are about 40 million amputees living in developing countries (Marino et al., 2015), and this number is expected to rise in the future (Ziegler-Graham et al., 2007). Many prosthetic devices have been proposed to raise the living standards of amputees by helping them restore their functional abilities, enabling them to perform daily chores, and return back to their work (Millstein et al., 1985).

Despite many potential benefits, a substantial percentage of people with upper-limb amputation prefer not to wear prostheses. In the literature, the mean rejection rates for the use of electric and body-powered prostheses are reported for the pediatric population as 35 and 45%, and for the adult population as 23 and 26%, respectively (Biddiss and Chau, 2007). Some of the reasons behind the low acceptance rate of body-powered hands are reported as slow movement, heavy-weight, inadequate grip force, limited functionality, inconvenience of harnessing, unnatural use, and discomfort (Biddiss and Chau, 2007).

Many research groups have investigated means to close the acceptance gap by orienting their studies to increase the dexterity and functionality of prosthetic hand devices. In both academic studies (Riillo et al., 2014; Naik et al., 2016; Wang et al., 2017) and commercial applications (Bebionic, 2022; Motion Control Inc., 2022; Ossur Inc., 2022; Ottobock, 2022), the most common means to control dexterous hand prosthesis is based on classifying surface electromyography (sEMG) signals recorded from different muscle groups and assigning a grip pattern to each class. Recently, some studies have also integrated different sources of data, such as mechanomiographic (MMG) biosignals (Kurzynski et al., 2016), near-infrared spectroscopy (NIRS) (Guo et al., 2017), and inertial measurement unit (IMU) (Kyranou et al., 2016), to improve the classification performance of multi-functional hand prostheses. Although such studies are aimed to make the amputees' life easier by enabling hand prostheses to have more functions, these devices demand long-training periods (Herle, 2016) stemming from their non-intuitive control interface and have not been shown to provide a viable solution for the high abandonment rate of prosthetic devices (Atkins et al., 1996).

To enable natural dexterity and an intuitive control interface for prosthetic hand devices, one of the prominent features of human neuromuscular system specialized to be competent at realizing various physical activities may provide a solution. In particular, most of the daily activities that require physical interactions with human hands are successfully performed because of the unrivaled capability of human adaptation. Such ability originates from predicting the type of the interaction and regulating the impedance of the limb based on the activity (Franklin and Milner, 2003; Franklin et al., 2003a,b, 2004; Popescu et al., 2003; Perreault et al., 2004). The impedance regulation of limbs is realized through the modulation of the contraction levels of antagonistic muscle pairs and reflexive reactions that contribute to neuromotor control to assist the stability of human-object interaction. All these abilities enable humans to actively and naturally perform activities of daily living (ADL). For instance, during tasks that require high precision (such as writing), humans raise the stiffness of their arm to guarantee the precise positioning against perturbations, while during interactions with soft/fragile objects, humans regulate their limbs to become more compliant in order to prevent damage to the object (Hogan, 2002).

The impedance modulation ability of humans has become inspiring in robotics. Along these lines, several studies on prosthetic devices have been conducted to imitate the stiffness regulation feature of humans, while physically interacting with their environment (Abul-Haj and Hogan, 1990a,b; Rao et al., 2010). Moreover, systematic human subject experiments have provided evidence that task-dependent impedance regulation improves human performance while using a virtual arm prosthesis (Blank et al., 2011, 2012, 2013). Recently, authors have proposed a variable stiffness transradial hand prosthesis (Hocaoglu and Patoglu, 2012, 2019b). Variable stiffness actuation (VSA) of this prosthesis is based on antagonistically arranged tendons coupled to nonlinear springs driven through a Bowden cable based power transmission. Unlike in the control based impedance modulation, VSA based prosthesis possesses high energy efficiency, since its actuators are not in use at all times to maintain the desired stiffness level. Furthermore, since the resulting stiffness of VSA is an inherent physical property of the device, it is valid over the whole frequency spectrum, including the frequencies over the controllable bandwidth of the actuators.

In this study, we propose, implement, and evaluate a natural human-machine interface for a variable stiffness transradial hand prosthesis to achieve tele-impedance control through sEMG signals. The mechatronic design of the transradial hand prosthesis, presented in Hocaoglu and Patoglu (2019a), employs a VSA based on the antagonistic actuation principle with quadratic springs and enables amputees to regulate the stiffness and position of the hand prosthesis independently. For the tele-impedance control of the variable stiffness transradial hand prosthesis, we benefit from sEMG signals generated during the muscular activity captured by biopotential electrodes, by means of which amputees can naturally be a part of the control architecture.

Our human machine interface is based on using four channels of sEMG signals responsible for controlling the position and impedance of the variable stiffness transradial hand prosthesis. In particular, as commonly done in the literature (Dalley et al., 2009; Bennett et al., 2016; Lenzi et al., 2016; Kim et al., 2019), the motion control of the hand prosthesis is regulated through intentional muscular activities generated at chest and shoulder mapped to the opening/closing of the fingers. However, in contrast to other interfaces, the stiffness of the prosthesis is regulated automatically based on the estimated stiffness of the *intact* muscle groups of the upper arm. As a result, while the proposed human machine interface requires the amputee to intentionally control the position of the VSA prosthesis, the stiffness regulation takes place automatically based on the instantaneous stiffness of the intact portion of the limb. Such an approach is advantageous since the impedance regulation takes place effortlessly from task to task or during the execution of a single task without requiring amputees' attention and diminishing their functional capability. Consequently, such an interface is easy to use, does not require long training periods, and does not interfere with the control of intact body segments. Furthermore, it has been pointed out in the literature that energetic interactions with the environment influence the determination of the impedance by the intact neuromuscular system (Franklin et al., 2004). Hence, regulating the prosthesis to mimic the impedance of an intact portion of the limb promises to be a more plausible control strategy than requiring the amputee to determine and control the proper impedance using dysfunctional muscles that lack such physical feedback, since these muscles are not physically coupled to the environment.

A preliminary study regarding tele-impedance control of variable stiffness transradial hand prosthesis has been presented in Hocaoglu and Patoglu (2012). This study significantly extends (Hocaoglu and Patoglu, 2012). To the best of the authors' knowledge, this study, along with Hocaoglu and Patoglu (2012), presents one of the first human-machine control interfaces for a VSA hand prosthesis. Furthermore, the human subject experiments presented in this study complement the ones in the literature (Blank et al., 2011, 2012, 2013; Hocaoglu and Patoglu, 2012), as physical interactions with the environment are enabled.

The contributions are summarized as follows: (i) A natural human-machine interface compatible with a variable stiffness transradial hand prosthesis is proposed. (ii) A muscle fatigue compensator responsible for the reference signal generation is designed and embedded into the proposed control architecture. (iii) The independent and simultaneous stiffness and position controls of variable stiffness hand prosthesis have been experimentally verified. (iv) Evidence is provided through human-subject experiments conducted over able volunteers on various tasks that the upper and lower arm impedance modulation display similar characteristics and impedances of both parts of the arm are modulated simultaneously for many tasks. (v) Experimental verification of the effectiveness of stiffness modulation and the need for short training periods have been demonstrated.

The rest of the article is organized as follows: Section 2 introduces sEMG-based tele-impedance control of the variable stiffness transradial hand prosthesis, details the construction of control references through sEMG based stiffness and position estimations, explains the independent control of the position and stiffness for the antagonist VSA, presents the compensation strategy used against muscle fatigue, and provides a set of experiments to verify the hypothesis that the stiffness modulation of the upper arm and the forearm are correlated. Section 3 experimentally verifies the independent control of the position and stiffness through the sEMG-based tele-impedance control of a VSA transradial hand prosthesis and provides evidence that the natural human-machine interface is an effective strategy in control. Section 4 concludes the article and discusses the limitations of the study.

## 2. Materials and Methods

This section conveys the technical details of our approach and presents the experimental verification of the effectiveness of each module in the approach. Accordingly, the tele-impedance control paradigm of a variable stiffness hand prosthesis is introduced in section 2.1, the modules constituting the paradigm are detailed, and experimental evaluations of each function in the modules are elaborated in sections 2.1.1 and 2.1.2. The compensation technique proposed for muscle fatigue due to co-contraction of the muscle pairs is circumstantiated through human-subject experiments in section 2.1.3. Moreover, the experimental validations of the methodologies to estimate the stiffness and position parameters through sEMG signals to be employed for the control of hand prosthesis are detailed in section 2.1.4. Section 2 is concluded with the experimental verification of the correlated stiffness adaptation of antagonistic muscle pairs.

### 2.1. sEMG Based Tele-Impedance Control of a Variable Stiffness Transradial Hand Prosthesis

Surface electromyography based tele-impedance controller is developed to control a VSA transradial hand prosthesis (Hocaoglu and Patoglu, 2019a). The transradial hand prosthesis features tendon driven underactuated compliant fingers that naturally adapt the hand shape to wrap around a wide variety of object geometries. Abiding by the definition of underactuation, two movable pulleys on the palm actuated by a VSA mechanism are assigned for the extension and flexion of the 12 DoF tendon-driven hand mechanism. Antagonistically arranged tendons of the prosthesis enable the modulation of the stiffness of the fingers and control of their position. Adaptation of the mechanical impedance of prosthesis based on changing physical conditions enables the amputee to perform various tasks with high dexterity.

**Figure 1:**
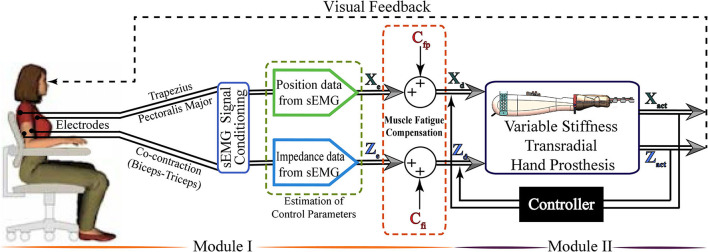
The control interface of the variable stiffness transradial hand prosthesis: In the first module, raw surface electromyography (sEMG) signals are measured from the upper arm and muscle groups placed under the chest and shoulder, sEMG signals are conditioned by a series of filters, and the position and stiffness references are estimated. The second module implements the position and stiffness control of the variable stiffness transradial hand prosthesis to follow the references estimated in the first module. Figure reproduced from Hocaoglu and Patoglu (2022).

Figure 1 presents an overview of the tele-impedance control architecture. The proposed control architecture consists of two modules. The first module handles the measurement of sEMG signals, their conditioning, and the estimation of reference values for the hand position and stiffness control. In the second module, the closed-loop motion controller [Proportional integral derivative (PID) controller] ensures the position and stiffness regulation of the VSA prosthetic hand based on the reference values estimated in the first module. In order to translate the meaning of normalized sEMG signals as physical references, namely position and stiffness references for the closed loop control system, one-to-one correspondence is assigned between the upper and lower limits of the normalized sEMG signals and angular position and stiffness limits of the fingers. Throughout the control, visual feedback and physical coupling provide information for the amputees to adapt their sEMG signals to match the task requirements.

Given that transradial upper extremity amputees lack the muscle groups responsible for hand and forearm motions, sEMG signals for the position control of the hand prosthesis are measured from the chest and the shoulder, while sEMG signals measured from the intact muscle pairs on the upper arm are used for the impedance control. Estimation of the hand position and stiffness from the sEMG signal involves modeling of hand motion/stiffness based on sEMG signals, empirical determination of model parameters for use in real-time control, and incorporation of fatigue compensation.

#### 2.1.1. Stiffness Estimation Through sEMG Signals

Muscle groups play a crucial role in the human body in terms of both the torque and impedance (stiffness and damping) modulation of a joint to properly interact with different environmental conditions. Particularly, impedance matching to the varying environment dynamics is carried out by means of the prominent features of muscles, such as regulation of co-contraction levels and reflex gains. The mechanical impedance of joints is an important parameter in the control of limbs under both static and dynamic conditions.

In the literature, many researchers have addressed the characterization of joint stiffness by focusing on multi-joint arm movements (Gomi and Kawato, 1996; Burdet et al., 2000, 2001). These studies are mainly focused on point-to-point reaching movements of subjects under perturbations and disturbance forces. The stiffness of the arm is estimated based on the relation between the deviations of the trajectories with respect to the undisturbed trajectories and the applied perturbation forces. Such methods are not viable for real-time applications, such as use with prosthetic limbs, as they require coupling users to a computer controlled manipulator. Index of muscle co-contraction around the joint (IMCJ) (Osu et al., 2002) approach is based on sEMG signals and provides a stiffness estimation technique that is feasible for real-time use. In this approach, the stiffness estimation is realized through the estimation of the co-contraction levels of antagonistic muscle groups. In the literature, the IMCJ method has been employed to reveal the mechanical characteristics of the musculoskeletal system (Hunter and Kearney, 1982; Basmajian and De Luca, 1985; Gomi and Osu, 1998).

Index of muscle co-contraction around the joint describes the working principle of antagonistic muscle groups around a joint through rectified sEMG signals and utilizes Equations (1)–(2) for stiffness estimation of the joint (Osu et al., 2002).


(1) $$\begin{array}{l} \tau =\overset{k}{\underset{i=1}{\sum }}[\kappa_{i}.agon\left(sEMG_{i}\right)-\lambda_{i}.anta\left(sEMG_{i}\right)] \end{array}$$

(2) $$\begin{array}{l} S=\overset{k}{\underset{i=1}{\sum }}[|\kappa_{i}|.agon\left(sEMG_{i}\right)+|\lambda_{i}|.anta\left(sEMG_{i}\right)] \end{array}$$

Here, *i* is the index that labels each muscle group, τ symbolizes the joint torque of the limb, while *agon*(*sEMG*) and *anta*(*sEMG*) denote the normalized muscular activity of the agonist and antagonist muscles, respectively. Symbols κ and λ capture the moment arms in charge of converting muscle activity to muscle tension responsible for generating muscle torque. The relation between the muscle torque and the muscle impedance (Murray et al., 1995; Kuechle et al., 1997; Gomi and Osu, 1998) is mapped to the correlation between the joint torque and the joint impedance (Hunter and Kearney, 1982; Gomi and Osu, 1998), leading to the joint stiffness estimates *S* *via* Equation (2), where κ and λ are estimated according to Equation (1).

In this study, Equations (1)–(2) were used to estimate the joint stiffness through a series of experiments as follows. Eight healthy volunteers (2 women, 6 men), who were students of Sabancı University participated in the experiments. Participants had no prior experience with the experimental setup. The participants did not report any sensory or motor impairment. All participants in all experimental studies presented in this article signed informed consent forms approved by the IRB of Sabancı University.

**Figure 2:**
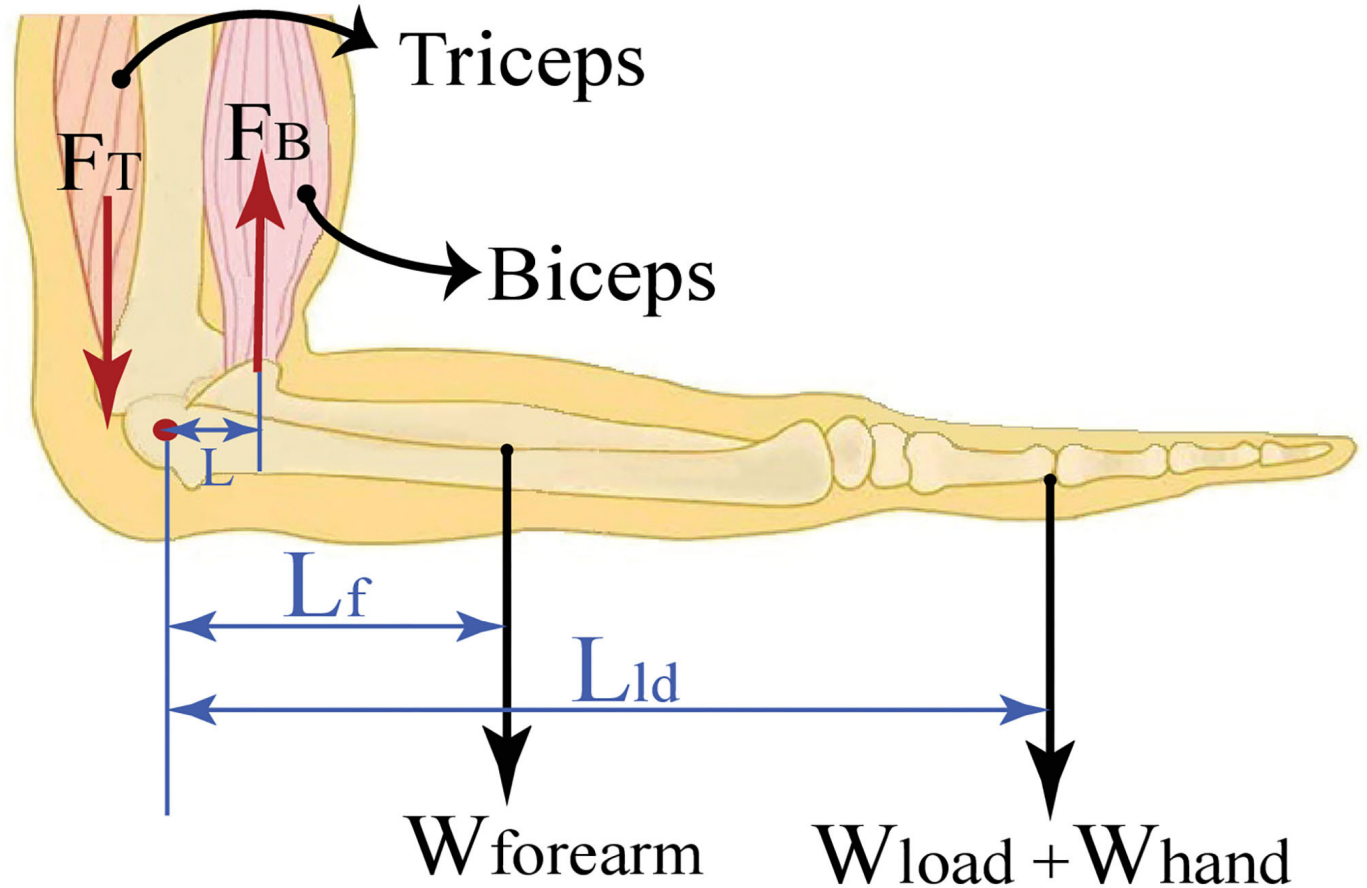
The biomechanical model with the pivot at the elbow joint and the elbow positioned at 90°.

The experimental task was to grasp a dumbbell while positioning the elbow at 90°, as shown in Figure 2. In particular, the forearm was configured horizontally, while the upper arm was kept perpendicular to the forearm with the palm was facing down. To maintain this configuration, the antagonistic muscle groups placed on the upper arm were isometrically contracted not to change the palm configuration and to exert appropriate forces to keep the joint angle at the desired value.

Participants started by lifting their forearm when their hand was free, and then the load was gradually increased using dumbbells of 0.5 kg, 1 kg, 1.28 kg, 2.26 kg, 2.76 kg, and 3.76 kg, respectively. Each condition was tested for 20 trials, where each trial lasted 20 s, on average.

The net torque applied at the elbow joint is calculated using the weight of the load *W*_*load*_ and the weight of the forearm *W*_*forearm*_ together with the moment arm corresponding to the load *L*_*ld*_ and the center of gravity of the forearm *L*_*f*_ with respect to the elbow joint.

**Figure 3:**
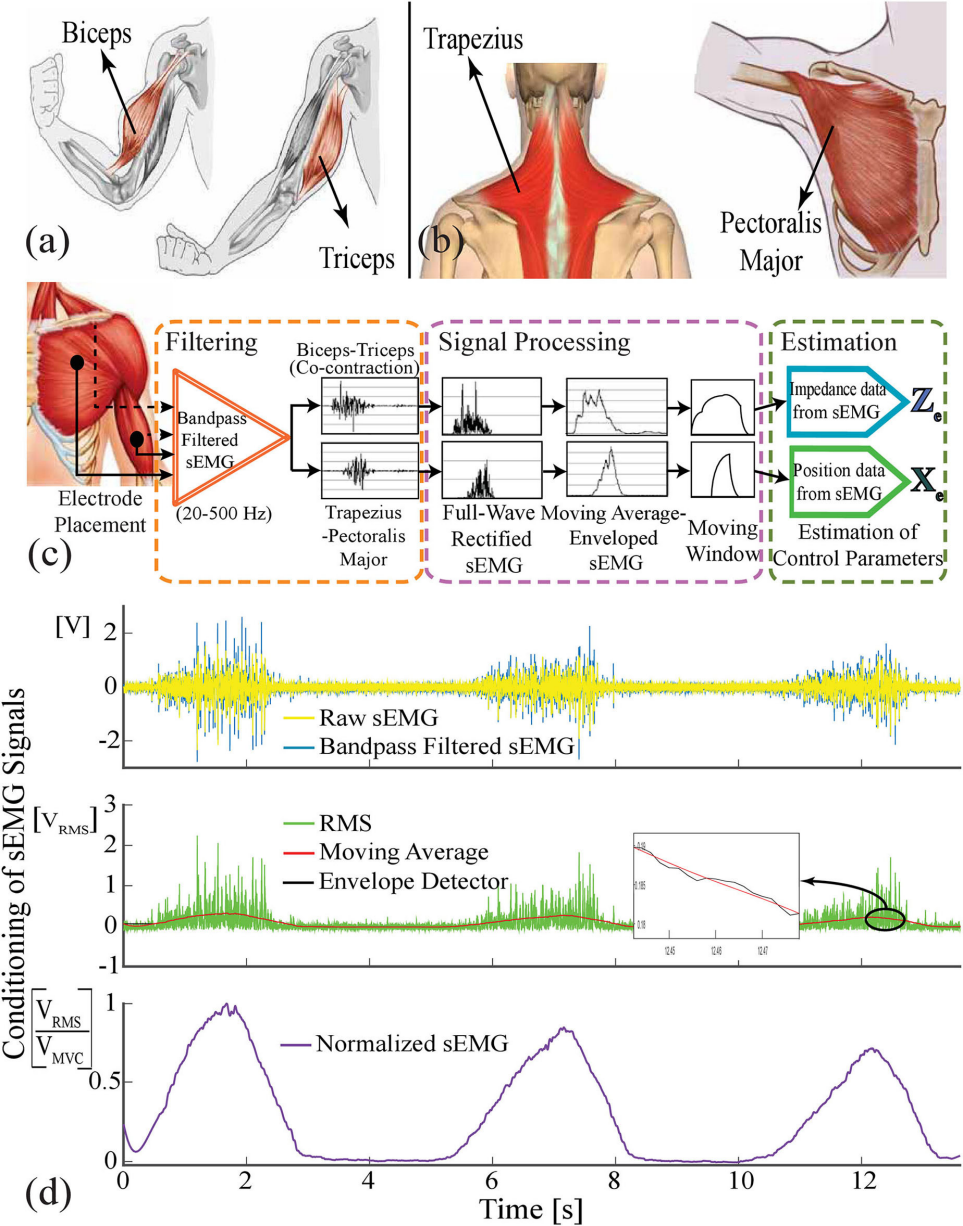
**(a)** Biceps and triceps muscles are responsible for the stiffness modulation. **(b)** Trapezius and pectoralis major muscles are employed for position regulation. **(c)** sEMG signal flow: Raw sEMG signals (yellow) are bandpass filtered (blue) and full wave rectified. Then, these signals are averaged using 0.5 second moving window and undesired ripples are omitted by means of envelope detection. **(d)** On the top graph, the raw sEMG data filtered against the inherent and environmental noises, and artifacts are represented with the blue signal. The second graph depicts the rectified (green), moving averaged (red), enveloped (black) sEMG signal. The bottom figure shows the normalized sEMG signal.

The antagonistic muscle pairs, biceps and triceps, responsible for generating the sEMG signals for the stiffness estimation are shown in Figure 3a. sEMG signals were measured by means of surface electrodes of an sEMG signal acquisition device with a sampling rate of 1 kHz. Raw sEMG signals were collected during the trials and conditioned by means of a full-wave rectifier, a 200 sample moving average filter, and an envelope detector. During the analysis, the first 500 samples of each trial were omitted from the experimental data to exclude signal outliers owing to initialization and motion artifacts.

The sequential processes of signal conditioning and reference estimation are illustrated in Figure 3c, while a sample signal extracted from a real-time experiment is presented in Figure 3d. After measuring the raw sEMG signals from two antagonistic muscle pairs responsible for stiffness and position controls of the hand prosthesis, they were filtered against inherent and environmental noises and motion artifacts utilizing a Butterworth band-pass filter with a frequency range of 20–500 Hz. After improving the signal-to-noise ratio of the sEMG signal, as illustrated in the purple dashed frame, the signal was full-wave rectified to correlate the behavior of muscles' contractions with physical variables, of position and stiffness references. As presented in Figure 3d, a moving window over a period of 0.5 s was employed to reveal the muscle's response against the task at hand. In addition, an envelope detector was utilized to filter out the ripples of the averaged signal. Such conditioning methods a play crucial role in revealing the relation between the joint torque and sEMG signals. Finally, the normalization of sEMG signals was carried out using the maximum voluntary contraction (MVC) of the participants, since these signals show different characteristics for each participant and vary their features at different time intervals. As depicted with the green dashed frame in Figure 3c, the signals were prepared for the estimation of the control parameters to correlate the finger and VSA kinematics with the normalized position and stiffness signals, respectively.

The parameters in Equation (1) were estimated using multiple linear regression by means of recorded data streams of *agon*(*sEMG*), *anta*(*sEMG*), and τ. For this linear model, the regression coefficients were obtained with 95% confidence bounds. The estimations for a subject are presented in Table 1. The quality of the estimation for all subjects was evaluated to be high, with *R*^2^ > 0.99 and *RMSE* < 0.03. Please note that this estimation procedure is repeated for each subject, before each use of the prosthetic hand.

**Table 1:** Estimated parameters of the stiffness model.

**κ**	**λ**	**R^**2**^**	**RMSE**
1.8612	1	0.9938	0.02941

#### 2.1.2. Position Estimation Through sEMG Signals

In order to achieve independent and simultaneous position and stiffness control, the overlap of sEMG signals corresponding to the stiffness reference with sEMG signals corresponding to the position reference has to be avoided. All muscle groups on the arm take part in the isometric contraction. Since sEMG signals measured from the upper arm are used to estimate stiffness reference, to avoid any overlap, pectoralis major and trapezius muscles placed in the chest and shoulder, shown in Figure 3b, are preferred for the position control of hand prosthesis. This selection ensures the independent location of muscle pairs responsible for stiffness modulation and position control, such that their activities do not directly affect each other.

The position of the underactuated variable stiffness hand prosthesis is controlled intentionally under visual feedback. The hand prosthesis in this study is a highly underactuated mechanism, as all fingers are connected to two main joints responsible for flexion and extension. VSA mechanism is actuated by two DC motors and controls the position and stiffness of the fingers. For the position control of the fingers, the required position reference signal is provided by the sEMG signals of the amputee. The exact positions of the fingers depend on the interaction between the prosthesis and the environment, as well as the position controller tracking the reference signal generated by the amputee. In this application, the precise estimation of position reference is not of critical importance, since the amputee can adjust the position of the prosthetic hand based on visual feedback. The position of the transradial prosthetic hand is controlled through a direct proportional relation between the intensity of sEMG signals with the desired joint angle of the fingers. Along these lines, the normalized sEMG signal responsible for position reference is expressed as ten discrete values, and each value is matched between the angular positions of the fingers in the rest and the fist states. In other words, different contraction levels of the responsible muscle groups indicate different ranges of normalized sEMG signals, and these values are mapped to different closure states of the fingers. For example, while the volunteer's normalized sEMG signals increase from 0 to 1 by contracting his/her muscles intentionally, the fingers start to rotate from their rest positions to reach a fist state. Since the required angular positions of the fingers to grasp the different geometric shapes of objects are different, volunteers adjust the contraction level of their muscles responsible for position control to different ranges based on visual feedback.

Another design parameter while constructing the position control references is the MVC percentage that is used for normalization. Instead of mapping 100% MVC to fully close/open the hand, a lower MVC can be set to decrease muscle fatigue to a great extent. In our study, the MVC level is selected as 70%, such that the position reference for the actuation of VSAs is calculated using the following normalized sEMG signal


(3) $$\begin{array}{l} sEMG_{normpos}=\frac{sEMG_{position}-sEMG_{bias}}{sEMG_{\%70MVC}} \end{array}$$

where *sEMG*_*normpos*_ denotes the normalized sEMG signals corresponding to position reference, *sEMG*_*position*_ represents the conditioned sEMG signals measured from pectoralis major and trapezius muscles, *sEMG*_%70*MVC*_ is 70% MVC of the responsible muscles, and *sEMG*_*bias*_ is the bias on the signal.

Another undesirable condition is the contamination of sEMG signals generated by pectoralis major responsible for the opening of the hand by electrocardiography (ECG) signals. ECG crosstalk effect is prevented from sEMG signals by avoiding the electrode placement in the contamination zone and by adding extra bias term to the sEMG signals until the ECG signal effect is suppressed.

#### 2.1.3. Compensation Against Muscle Fatigue

Muscle fatigue can be defined as a decline in the muscle strength to generate force, that is, a decrease in the sEMG amplitude as a result of the reduction in active muscle fibers during ceaseless muscle activity (Al-Mulla et al., 2011). The reason for muscle fatigue encompasses the metabolic, structural, and energetic alternations in muscles owing to insufficient oxygen level, inadequate blood circulation responsible for supplying nutritive substances, and also decrease in the efficiency of the nervous system (Merletti et al., 2005).

Myoelectric signals collected on the surface of the skin can be used for real-time monitoring of muscle fatigue (De Luca, 1984). This method is commonly preferred since it can provide uninterrupted data recordings related to muscle fatigue with a non-invasive technique, even though this method has certain disadvantages, such as the difficulties associated with exact positioning of surface electrodes on desired muscles and undesired cross-talk of the myoelectric signals with the neighboring muscles. A large number of studies have been performed to establish signal-based quantitative criteria to characterize muscle fatigue under static and dynamic tasks. Along these lines, numerous classical and modern signal processing methods have been established for sEMG-based muscle fatigue evaluation (Cifrek et al., 2009).

In this study, we rely on a time-domain root-mean-square (RMS) feature of sEMG signals to compensate for the fatigue effect (Bilodeau et al., 2003). In particular, during the use of the prosthetic device, the muscle performance decreases as a function of use time; as muscle fatigue increases, the sEMG-based stiffness reference estimates deteriorate. RMS feature based fatigue compensation estimates the decrease in sEMG signal power as a function of use time and introduces a compensation factor to counteract this fatigue.

**Figure 4:**
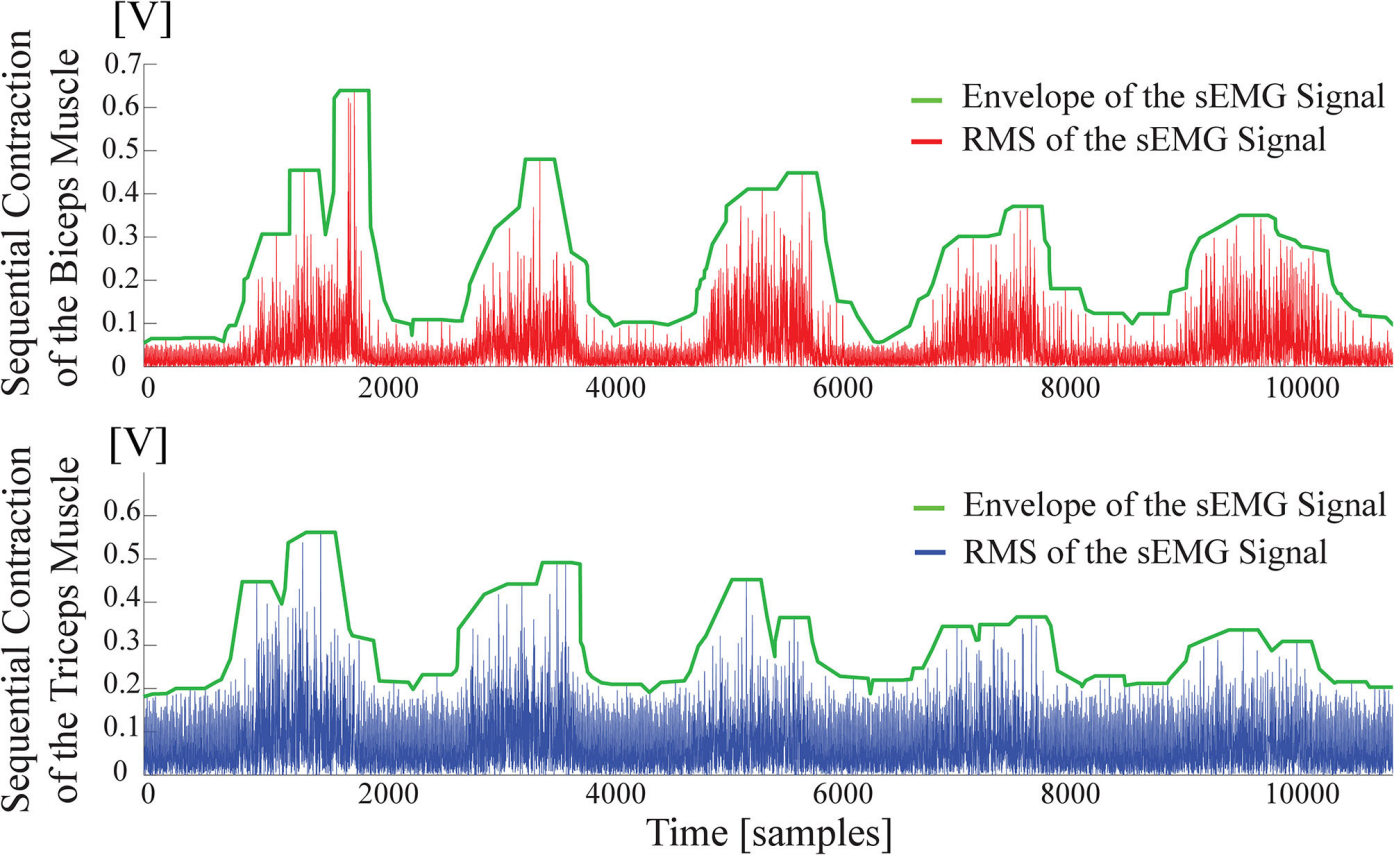
Surface electromyography signal features capturing the average fatigue characteristics of biceps and triceps muscles.

Muscle fatigue compensation is activated in the control loop when a threshold is exceeded. A moving average window of 2,000 samples runs to check the presence of the consecutive contractions, by comparing the average level of enveloped sEMG signal under the moving window with the threshold. The threshold commissioned for activation of the fatigue compensation is empirically determined as about 20% MVC and varies slightly among volunteers. Figure 4 illustrates muscle fatigue captured by sEMG signals when a volunteer repeatedly co-contracts her muscles within 5 s. In the figure, the green line presents the envelope of RMS of sEMG signals and the decrease of signal power can be observed.

To estimate the fatigue characteristics from sEMG signals, an experiment is conducted where a volunteer is requested to realize sustained isometric contractions periodically. In each session, the volunteers are instructed to perform isometric contractions five times. The experiment includes 10 sessions with each session including 5 trials lasting for 30 s.

The fatigue behavior of the individual is extracted from the sEMG data through three sequential signal conditioning stages. First, the raw sEMG signals are band-pass filtered with a frequency band between 20-500 Hz to remove undesired signals due to electronic noise, motion artifacts, ECG cross-talk, and power-line interference. Second, the filtered sEMG signal is normalized with the MVC of the volunteer. Finally, the RMS of the sEMG signal is calculated.

**Figure 5:**
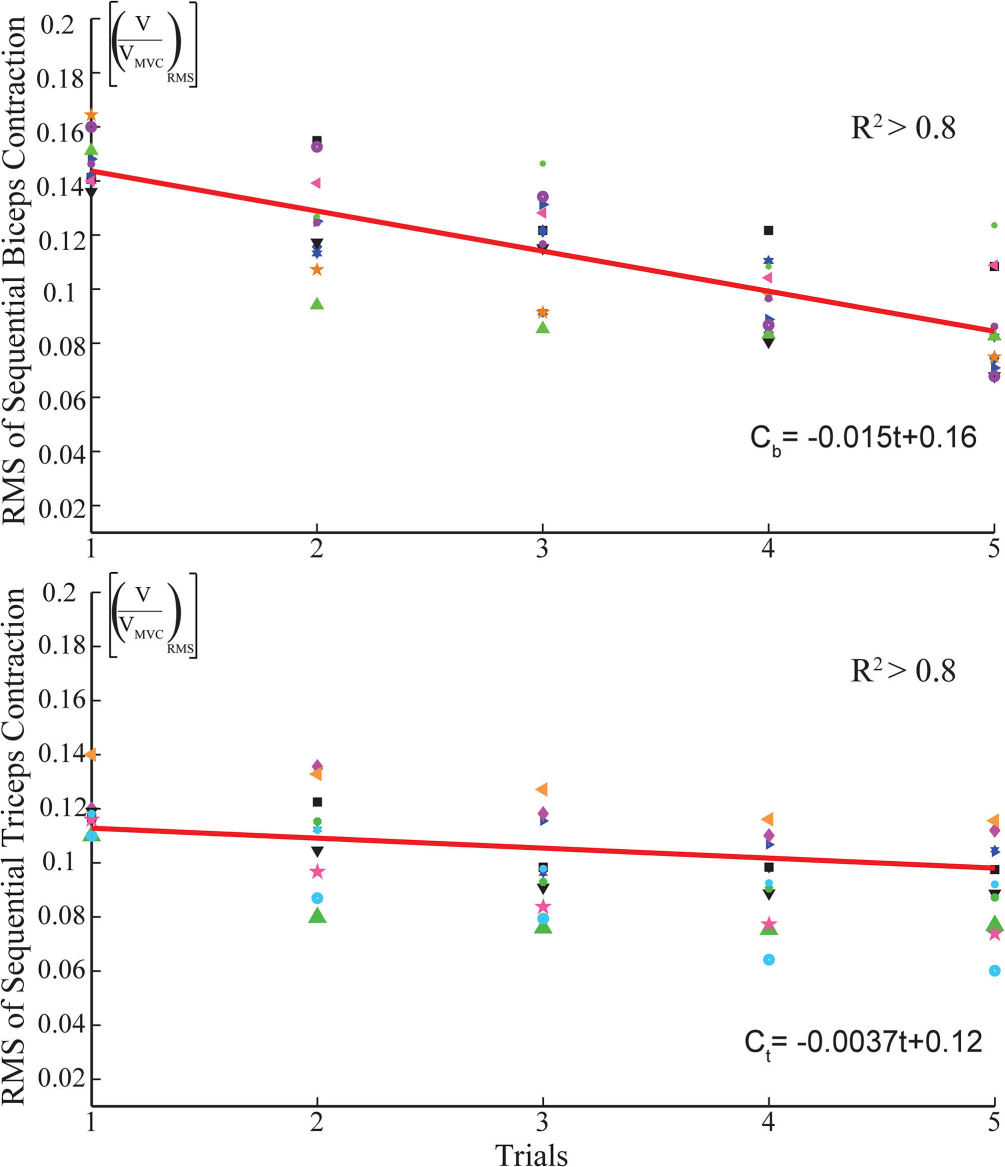
Linear fits capturing the average fatigue characteristics of biceps and triceps muscles.

Figure 5 presents sample results characterizing the fatigue observed on the biceps and triceps muscles as a function of the time. The muscle fatigue behavior during a session, i.e., the RMS of each contraction (trial) in a session is represented by the same geometric symbol in Figure 5. Each session has its own respective symbols to help with the identification of muscle fatigue. In particular, the star symbol represents a consecutive contraction, namely the trial, of the subject in a session. The average of 10 sessions is represented by a dark blue star icon in the graph. Linear fits, as presented in Figure 5, are sufficient to capture the time dependent fatigue characteristics embedded in this data set, as evidenced by the good quality of curve fits (*R*^2^ > 0.8). Once these linear estimates are at hand, they can be incorporated in the stiffness reference estimation as a feed-forward compensation term denoted by *C*_*fi*_ in Figure 1. Unlike the impedance modulation, position control typically does not require sequential contractions; hence, the muscle fatigue is neglected during position regulation, that is, no feed-forward compensation is performed for the position control by setting *C*_*fp*_=0 in Figure 1.

#### 2.1.4. Position and Stiffness Regulation With Antagonist VSA

**Figure 6:**
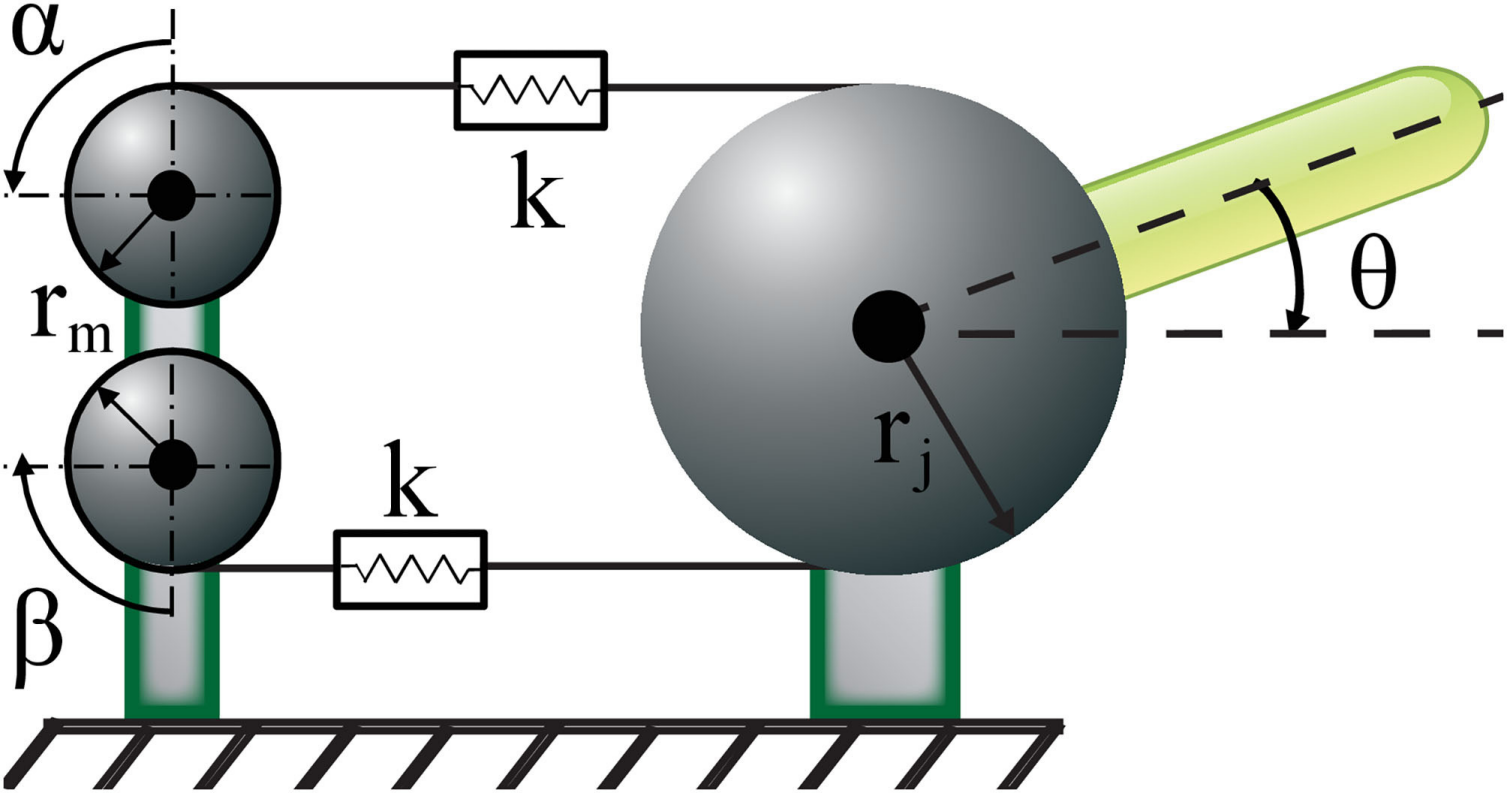
Schematic model of an antagonistically driven VSA, where *k* represents the nonlinear springs.

Given the sEMG based position and stiffness reference estimation and fatigue compensation processes, the second module of the interface is a controller that ensures tracking of these references by the VSA prosthetic hand. In particular, the position and stiffness of the VSA are controlled through position control of Bowden cables driven by two geared DC motors. Figure 6 presents a schematic representation of the VSA, where α and β denote the angular position of DC motors, while *S* and θ represent the joint stiffness and angle, respectively.

Under quasi-static conditions (English and Russell, 1999; Migliore et al., 2007), the angular position of DC motors α and β for a given reference position θ_*r*_ and stiffness *S*_*r*_ can be calculated as


(4) $$\begin{array}{l} \alpha =\left(S_{r}-2br_{j}^{2}\right)/4ar_{m}r_{j}^{2}+\left(r_{j}/r_{m}\right)\left(\left(\tau_{load}/S_{r}\right)-\theta_{r}\right) \end{array}$$

(5) $$\begin{array}{l} \beta =\left(S_{r}-2br_{j}^{2}\right)/4ar_{m}r_{j}^{2}-\left(r_{j}/r_{m}\right)\left(\left(\tau_{load}/S_{r}\right)-\theta_{r}\right) \end{array}$$

where *r*_*m*_ represents the radius of the pulleys attached to the geared DC motors, *r*_*j*_ is the radius of the drive pulley, *a* and *b* are the parameters that characterize the expanding contour cam as detailed in Hocaoglu and Patoglu (2019a), while the external torque applied to VSA is denoted by τ_*load*_. When control references belonging to joint position and stiffness are estimated through sEMG signals, desired motor positions are computed according to Equations (4)–(5) with τ_*load*_ = 0 and motors are motion controlled to these values under real-time control.

### 2.2. Verification of Correlated Stiffness Adaptation of Antagonistic Muscle Pairs

The stiffness of the prosthesis is regulated automatically based on the estimated stiffness of the *intact* muscle groups of the upper arm. This control strategy, in which the prosthesis mimics the impedance of an intact portion of the limb, relies on the assumption that the impedance of the upper and lower arm change similarly, during energetic interactions with the environment.

We have conducted a series of experiments to test the validity of this assumption. During these experiments, the stiffness of both the forearm and upper arm of participants is estimated through the sEMG signals collected from the relevant antagonistic muscle pairs, using the technique detailed in section 2.1.1. Hence, during these experiments, the stiffness estimations of the upper and lower arm are performed based on sEMG signals and the load applied. Eight healthy volunteers took place in the experiments. The experiments were conducted for two tasks: i) a load bearing task and ii) interaction with the various objects with different impedance characteristics.

**Figure 7:**
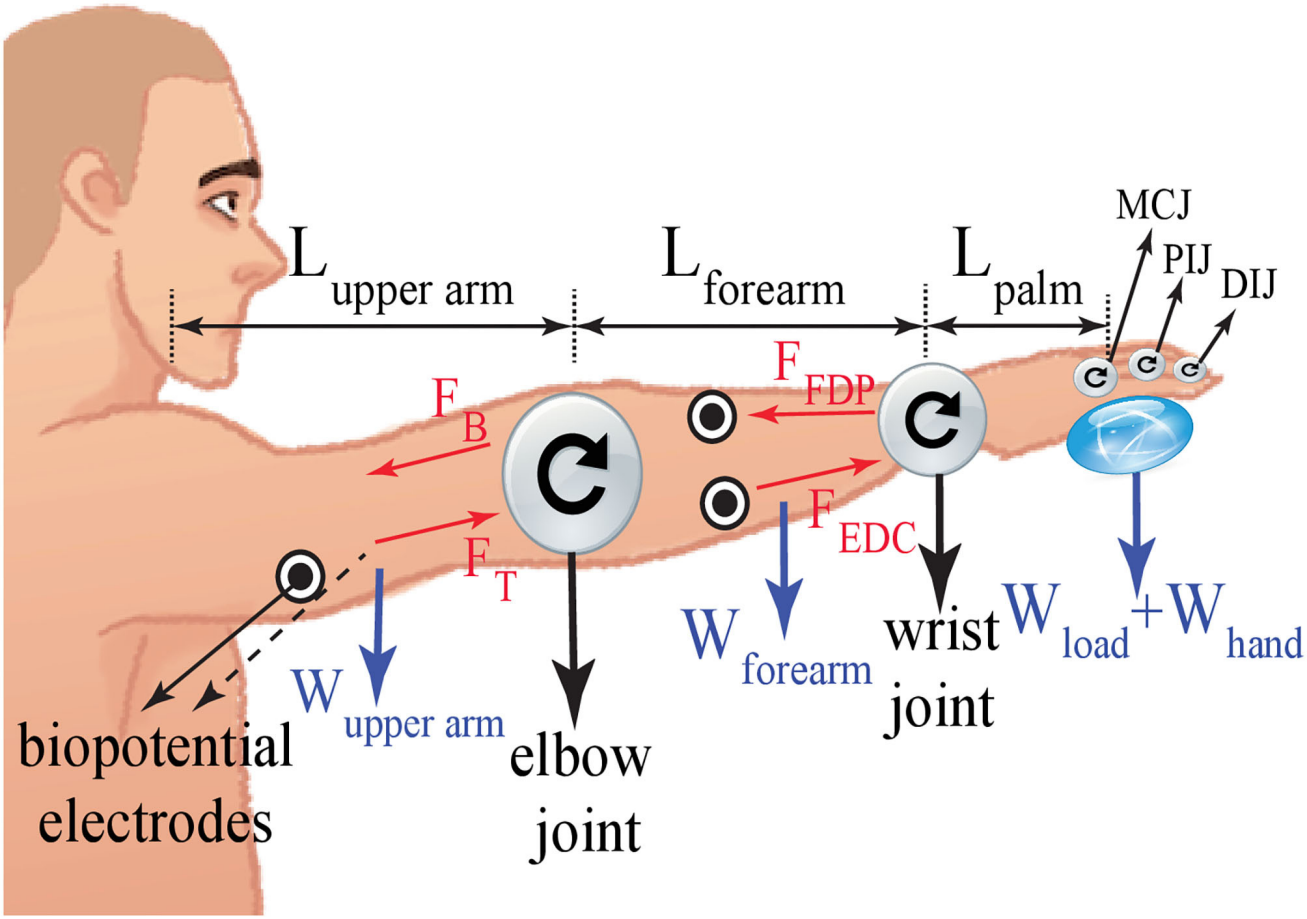
The biomechanical model with the pivots at the wrist and elbow joints, while keeping the arm straight and forward.

The first task aims to observe resistance of the hand, forearm, and upper arm against displacement stemmed from the weight of an object with respect to the arm's normal posture. During the first task, participants were asked to keep their arms straight and forward as depicted in Figure 7. The stiffness of the upper arm and forearm were estimated as the load at the hand was increased incrementally. In particular, the load was gradually increased from no load to 0.5, 1, 1.5, 2, and 3 kg. Each task was repeated 5 times and each trial lasted about 8 s. Sufficient rest time was provided between consecutive trials to prevent muscle fatigue.

Stiffness estimation was performed as detailed in section 2.1.1. sEMG signals were collected from the antagonistic muscle groups of flexor digitorum profundis and extensor digitorum at the forearm, and biceps and triceps at the upper arm. Since the stiffness of both the upper arm and forearm was estimated, two separate biomechanical models were derived around the elbow and wrist joints, respectively. The net torque applied on the joints was calculated considering the weight of the grasped load *W*_*load*_, the hand *W*_*hand*_, the forearm *W*_*forearm*_, and the upper arm *W*_*upperarm*_ together with their respective moment arms.

**Figure 8:**
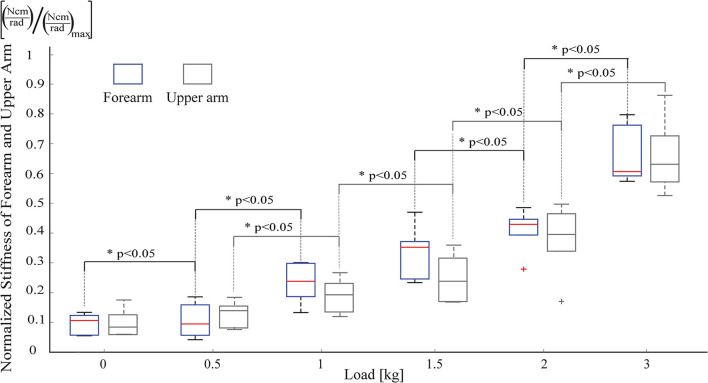
Stiffness estimates from the forearm and the upper arm of participants, while resisting against increasing loads.

Figure 8 depicts the estimated stiffness levels at the forearm and the upper arm, under various loading conditions. As expected, as the load is increased, the stiffness of both the upper arm and the forearm increases. As presented in Figure 8, the change in stiffness levels is statistically significant between almost all pairs of loading conditions (with *p* < 0.05). More importantly, one can observe from these plots that the stiffness increase in the forearm and the upper arm are strongly correlated, and there exists no statistically significant difference between the forearm and the upper arm stiffness levels for each loading condition, for the load bearing task.

The second task tested the adaptation of the upper arm and the forearm impedance levels while interacting with several objects, to mimic common interactions taking place during ADL. In particular, participants started at a rest position, lifted their arm, reached toward the object, grasped it, held it for a while, released it on the table, and returned to their initial configuration. Three different object types were included in the experiment: A sponge, an empty glass, and a water-filled glass were employed for different impedance requirements. Each object was grasped five times and each trial took about 7 s. Sufficient rest time was provided to volunteers between sequential trials to prevent muscle fatigue.

The objects were selected such that their manipulation emphasized different control strategies, ranging from precise motion control to robust force control. Due to the complexity of the task that involved multiple sub-movements, participants' stiffness levels went over continual changes throughout the trials. To quantitatively characterize the correlation between the stiffness of the upper arm and the forearm for each subject, a moving average filter is used to extract average stiffness variations from the instantaneous estimates. Table 2 presents the Pearson's correlation coefficient for these time series comparisons for each subject. In this table, the concordance correlation coefficients have large values of about 0.8, providing strong evidence that the impedance adaptation behavior of the upper arm and the forearm were in good agreement with each other throughout the complex manipulation task.

**Table 2:** Pearson's correlation coefficient between the stiffness modulation of the upper arm and the forearm muscles.

**Subject**	**Sponge**	**Glass**	**Water filled glass**
Subject 1	0.9246	0.9914	0.9447
Subject 2	0.9771	0.9180	0.9000
Subject 3	0.9863	0.9134	0.9222
Subject 4	0.9000	0.9234	0.9216
Subject 5	0.9000	0.9260	0.9715
Subject 6	0.9685	0.9158	0.9363
Subject 7	0.9062	0.9892	0.9148
Subject 8	0.9611	0.9425	0.9775

## 3. Experimental Evaluation of the Natural Control Interface

In this section, we present the evaluation of the integrated system, where stiffness and position estimation modules and sEMG based control are utilized simultaneously. We verify the feasibility and effectiveness of the proposed sEMG based human-machine interface that automatically modulates the impedance of VSA prosthetic hand while users intentionally control the hand position. For this purpose, we present two experiments where the independent control of hand position and stiffness were demonstrated. Section 3.1 details the experimental set-up and procedure used to verify the effectiveness and utility of the proposed sEMG-based control architecture to control VSA prosthesis. Section 3.2 presents the position and stiffness control tasks and experimental procedures used in the experiments. Section 3.3 details the results of the experiment, while Section 3.4 provides illustrative experiments where volunteers perform various grasps for different stiffness and geometric shape of objects.

### 3.1. Experimental Setup

Human-subject experiments on able volunteers were conducted using the VSA transradial hand prosthesis detailed in Hocaoglu and Patoglu (2019a). In the current design, the prosthesis does not feature a thumb but relies on passive elastic support that can counteract finger forces. This decision is intentional and helps to keep the system and the controller simple. Our experiences with the volunteers indicate that the passive support is adequate for implementing a wide variety of functional grasps.

**Figure 9:**
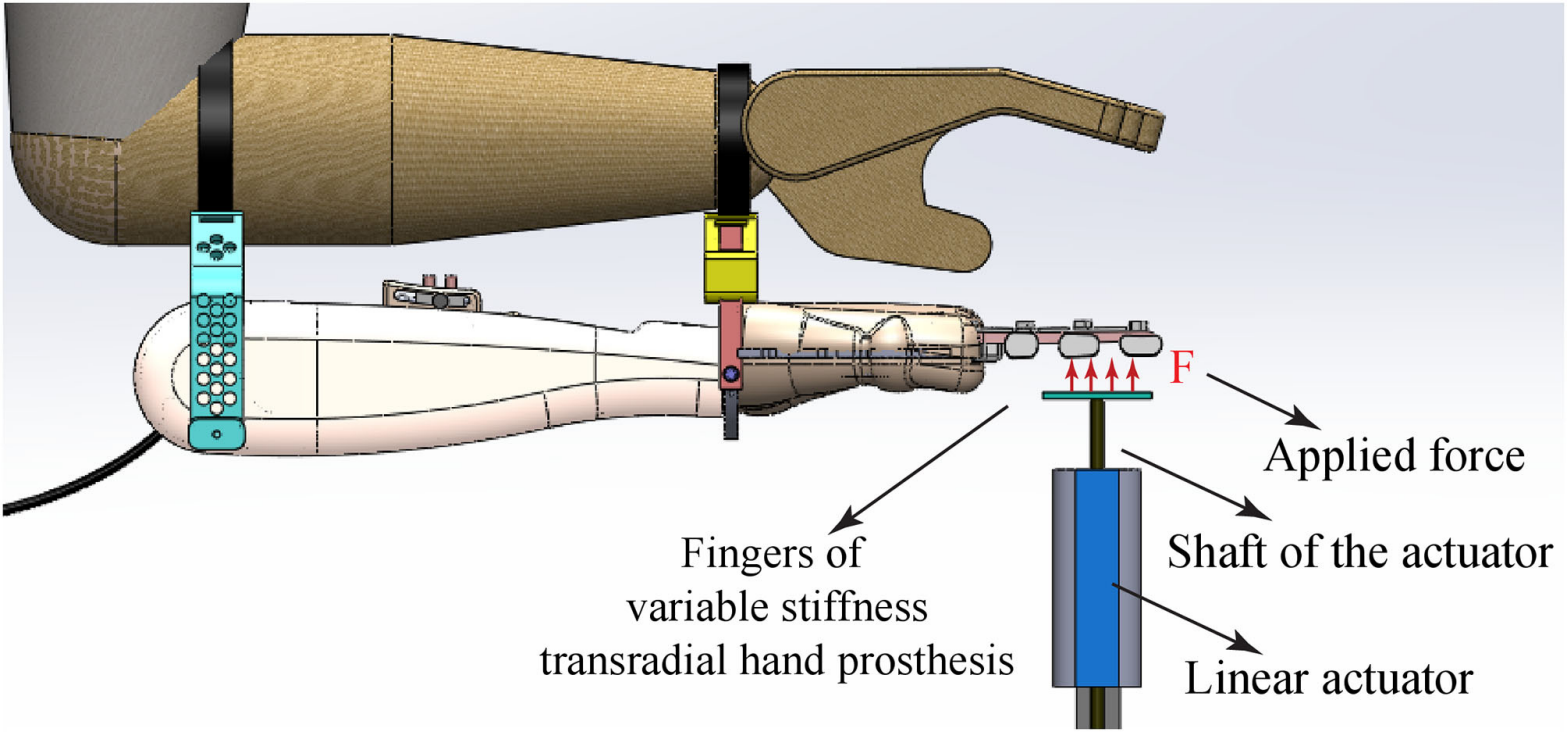
Schematic representation of the experimental setup: The linear actuator is used to apply controlled forces to the fingers and to measure their deflection under position and stiffness modulation tasks. Figure reproduced from Hocaoglu and Patoglu (2022).

Throughout the experiments, sEMG signals were collected from biceps and triceps muscles for stiffness modulation and from trapezius and pectoralis major muscles for position control using a data acquisition system with active electrodes. Stiffness and position references were estimated as discussed in section 2.1 and fed to the tracking controller that controlled two geared Direct Current (DC) motors under Proportional Derivative (PD) control in real-time at 500 Hz through a PC based Data Acquisition (DAQ) card. The robust position controller of each DC motor enables the system to achieve the desired joint position and joint stiffness settings as computed according to Equations (4)–(5) by properly actuating the angular positions (α and β) of the motors. A direct drive linear actuator combined with a precision position encoder was placed under the fingers of the hand prosthesis, as shown in Figure 9 to render forces and measure finger deflections. During the experiments, the gravitational force acting on the linear actuator was compensated with a counter mass, while the linear actuator was force controlled.

### 3.2. Experimental Procedure

Experiments were conducted to test the independent control of the position and the stiffness of the prosthetic hand under sEMG based tele-impedance control interface.

Throughout the experiments, the transradial hand prosthesis was worn by the volunteers, such that interaction forces with the environment provided direct power coupling with the volunteer. Note that such feedback is a crucial part of any prosthesis; however, has been neglected in Virtual Reality (VR) based studies (Blank et al., 2011, 2012, 2013).

Five healthy volunteers took place in the experiments. The prosthesis was worn parallel to the volunteers' lower arm, such that consistent placement of the prosthesis was ensured for proper hand-eye coordination.

The experiments were composed of two tasks with 10 repetitions for each condition of each task. During the first task, the position of the VSA hand prosthesis was kept constant at 0° while the stiffness of VSA was adjusted by the volunteers to five distinct stiffness values that correspond to a low, three intermediate, and a high stiffness level for the fingers. The stiffness of the fingers was experimentally determined by applying a linearly increasing force to flex the fingers and recording their deflection.

During the second task, the stiffness of the VSA hand prosthesis was kept constant at its intermediate level by the volunteers, while the position of the VSA was adjusted by the volunteers to three distinct position values that correspond to low, intermediate, and high flexion of the fingers. The position of the fingers was determined by recording the position of the linear actuator under zero force control, while the stiffness of the fingers was determined by applying a constant force to resist the flexion of the fingers at their equilibrium position and recording the resulting deflection.

### 3.3. Experimental Results

**Figure 10:**
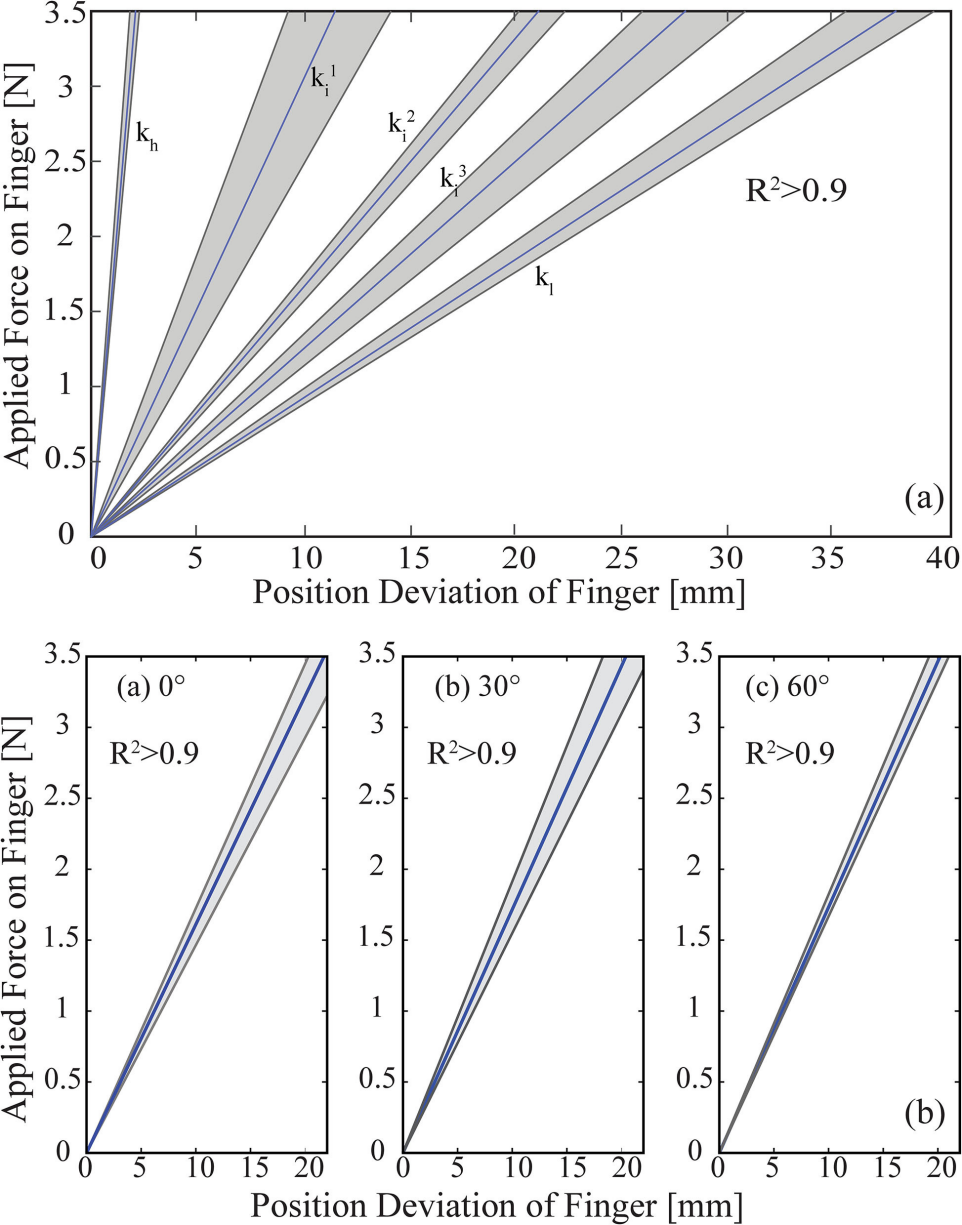
**(a)** Stiffness modulation of hand prosthesis through sEMG based tele-impedance control. **(b)** Position control of hand prosthesis through sEMG based tele-impedance control. In the figures, gray zones present the results of each trial, and the blue lines represent the average value of ten trials. Figure reproduced from Hocaoglu and Patoglu (2022).

Figure 10a presents the experimental results for the case when the volunteers adjusted the VSA stiffness to five distinct values that correspond to a low, three intermediate, and a high stiffness level for the fingers, while the finger positions were kept constant. In particular, shaded regions represent all the linear fits recorded for 10 trials, while the dark line represents their mean. The slopes of these lines indicate that the high, three intermediate, and the low stiffness for the fingers were *k*_*h*_ = 1.7 N/mm, $k_{i}^{1}$ = 0.3 N/mm, $k_{i}^{2}$ = 0.16 N/mm, $k_{i}^{3}$ = 0.12 N/mm, and *k*_*l*_ = 0.091 N/mm, respectively. The *R*^2^ values for these linear fits are higher than 0.97.

Figure 10b presents the experimental results for the case when the volunteers kept the VSA stiffness at an intermediate level, while the finger positions of the fingers were regulated by the volunteers to 0°, 30°, and 60°, respectively. Once again, the shaded regions represent all the linear fits recorded for 10 trials, while the dark line represents their mean. The slopes of these lines indicate that the stiffness levels of the fingers were $k_{0^{˙}}$ = 0.16 N/mm, $k_{30^{˙}}$ = 0.17 N/mm, and $k_{60^{˙}}$ = 0.17 N/mm, respectively. The *R*^2^ values for these linear fits are higher than 0.98.

The fingers' response shown in Figure 10 closely matches the characteristics of human fingers, as presented in Howe et al. (1985). The characterization results are also compatible with the results presented in Matsuoka and Afshar (2004), as flexion/extension movements performed by an anatomically human-like robotic index finger necessitate a similar amount of muscle forces.

Experimental results indicate that the sEMG based impedance controlled VSA hand prosthesis possesses very similar performance to the case with an external reference generator, as presented in Hocaoglu and Patoglu (2019a). In particular, volunteers were able to modulate their stiffness levels to the minimum and maximum stiffness limits of the prosthetic hand, as well as to various intermediate ranges, by means of the sEMG based tele-impedance control. These results provide evidence that the stiffness and position of the transradial hand prosthesis can be controlled independently by users, with high repeatability.

### 3.4. Illustrative Experiments and Evaluations

Given that only the position and the stiffness of the drive tendon can be directly regulated by the volunteers, in general, the resulting position and the stiffness of the fingers depending on the interaction. To test the usefulness of the sEMG based tele-impedance control interface of the variable stiffness transradial hand prosthesis, the device was attached to six volunteers, as shown in Figure 9, and the volunteers were given control of the position and stiffness of the prosthesis through the sEMG based tele-impedance controller. In particular, sEMG signals measured from the surface of the upper arm were used to automatically adjust the stiffness level of the prosthesis to that of the upper arm, while the position regulation was intentionally controlled by the volunteers by moving their shoulder muscles.

**Figure 11:**
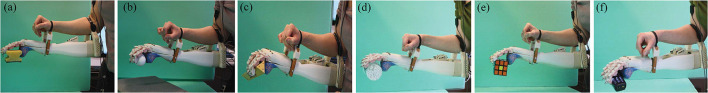
Demonstration of variable stiffness transradial hand prosthesis performing various grasps with the sEMG based tele-impedance control interface, while interacting with **(a)** a deformable object, **(b)** a fragile object, **(c)** a triangular rigid object, **(d)** a cylindrical rigid object, **(e)** a square rigid object, and **(f)** a rectangular rigid object.

The volunteers were instructed to grasp 16 objects with a broad array of geometries (e.g., cylindrical, square, oval, or unstructured) and elasticities (e.g., stiff, soft), as shown in Figure 11. In Figure 11a, a deformable sponge, in Figure 11b, a fragile raw egg were grasped by the volunteers with this natural control interface, without damaging the objects. In Figures 11c–f, rigid objects with various shapes were grasped by the volunteers using different stiffness levels. Videos demonstrating several illustrative grasps by a volunteer are available at https://youtu.be/fGFIKSSmtDg. The average time required to grasp and release the objects in the video is calculated as 1.218 ± 0.564 s and 0.819 ± 0.48 s, respectively. The time elapsed to make a fist is about 2 s. The commercial prosthetic hand devices (Ossur Inc., 2022) present quite the same grasping performance as the proposed variable stiffness hand prosthesis.

The proposed tele-impedance controller interface emphasizes simplicity, ease of use, and adaptability; hence, implements automatic modulation of prosthetic hand stiffness to match that of the upper arm, while intentional control of the position of the underactuated prosthetic hand is left to the user. Under the observation that humans tend to modulate the impedance of their limb as a whole while executing different tasks (as shown in section 3), the tele-impedance controller implemented for the prosthesis automatically modulates the stiffness of the hand to match that of the intact part of the arm. Automatic stiffness modulation increases the dexterity of the prosthetic hand, without introducing complexity to the human control interface.

Successful interactions with the prosthesis depend on the amputee making proper decisions on how to interact with the object under visual feedback and physical coupling. Our extensive experiments with six healthy volunteers indicate that humans are very skillful at learning how to interact with the environment with such a device under the proposed sEMG based natural control interface. All volunteers were able to adapt to the device on average in 3.2 ± 1.3 min and successfully complete the required manipulation tasks without any prior training. Furthermore, it has been observed that the stiffness modulation property is effective in increasing the performance of the transradial prosthesis.

Volunteers suffer from the high complexity of the controller when intentional control of both the stiffness and the position of the device is left to the user. During our tests, volunteers indicated a strong preference for the automatic impedance adjustment property. Furthermore, it has been observed that volunteers are more successful at interactions when the impedance of the prosthesis is automatically adjusted.

## 4. Discussion

Tele-impedance control of a VSA prosthetic hand is implemented through stiffness and position estimates decoded from sEMG signals of muscle groups embedded in the upper arm, chest, and shoulder. In particular, the IMCJ method is used to estimate the stiffness of the intact upper arm through agonist/antagonist muscle pairs, while shoulder/chest muscles are employed to estimate position references. Then, these stiffness and position estimates are used to control a VSA prosthetic hand.

The feasibility of tele-impedance control through the proposed human machine interface is demonstrated with two human subject experiments, where the position and the stiffness of the VSA prosthetic hand were successfully modulated. The results demonstrate that both position and stiffness estimations from sEMG signals are adequate for the control of a VSA transradial hand prosthesis.

Variable stiffness actuation hand prosthesis together with the proposed control interface necessitates less effort and concentration to control and is easier for the amputee to learn to use. Impedance modulation takes place naturally from task to task or while performing a task, i.e., ADL, without requiring amputees' attention, and this feature improves the performance of the prosthesis while interacting with unstructured environments.

The human subject experiments presented in this study have been performed on able volunteers. Our future studies include validation of the results on amputees. While special attention has been given to the selection of muscle groups used for sEMG based control, such that the same muscle groups can be recruited for transradial amputees, possible complications may arise in amputees due to muscle weakness stemming from infrequent use of the remnant limbs in ADL. Along these lines, the lack of verifications with amputees is a limitation of this study.

Second, the position and impedance regulation experiments have been performed on carefully controlled environments, as tight control of the experimental conditions was necessary to ensure that the results are statistically reliable with no confounding factors. Furthermore, sEMG based position and stiffness models can only provide rough estimations of human behavior. This study aims to provide easy control of a variable stiffness prosthetic hand instead of actually copying human behavior. While the same level of accuracy with the human arm may not be achieved in real-life use, our experiments with healthy volunteers presented in section 3.4 provide evidence that the level of control that can be achieved during grasping different objects is adequate to provide the required level of performance.

As part of future study, different control modes may be introduced to the system, for instance, to avoid the need for voluntary contraction once an object is successfully grasped. Additional feedback pathways, such as vibrotactile feedback, may be added to the system to decrease the need for visual feedback during grasping. Furthermore, sEMG related coefficients can be identified online to avoid the need for calibration of device.

## Data Availability Statement

The raw data supporting the conclusions of this article will be made available by the authors, without undue reservation.

## Ethics Statement

The studies involving human participants were reviewed and approved by the University Research Ethics Council of Sabancı University. The patients/participants provided their written informed consent to participate in this study.

## Author Contributions

EH was responsible for the design and experimental evaluation of the natural control interface of the variable stiffness hand prosthesis. VP conceived and supervised the study. Both authors contributed to the editing and scientific presentation of the article.

## Funding

This work has been partially supported by Tubitak Grants 219M586, 111M186, 115M698 and Marie Curie IRG Rehab-DUET.

## Conflict of Interest

The authors declare that the research was conducted in the absence of any commercial or financial relationships that could be construed as a potential conflict of interest.

## Publisher's Note

All claims expressed in this article are solely those of the authors and do not necessarily represent those of their affiliated organizations, or those of the publisher, the editors and the reviewers. Any product that may be evaluated in this article, or claim that may be made by its manufacturer, is not guaranteed or endorsed by the publisher.
